# Development of Wrist Separated Exoskeleton Socket of Myoelectric Prosthesis Hand for Symbrachydactyly

**DOI:** 10.34133/cbsystems.0141

**Published:** 2024-07-15

**Authors:** Yuki Inoue, Yuki Kuroda, Yusuke Yamanoi, Yoshiko Yabuki, Hiroshi Yokoi

**Affiliations:** ^1^ Graduate School of Informatics and Engineering, The University of Electro-Communications, Tokyo, Japan.; ^2^ Center for Neuroscience and Biomedical Engineering, The University of Electro-Communications, Tokyo, Japan.

## Abstract

In recent years, the functionality of myoelectric prosthetic hands has improved as motors have become smaller and controls have become more advanced. Attempts have been made to reproduce the rotation and flexion of the wrist by adding degrees of freedom to the wrist joint. However, it is still difficult to fully reproduce the functionality of the wrist joint owing to the weight of the prosthesis and size limitations. In this study, we developed a new socket and prosthetic hand control system that does not interfere with the wrist joint motion. This allows individuals with hand defects who previously used prosthetic hands with fixed wrist joints to freely use their remaining wrist functionality. In the pick-and-place experiment, where blocks were moved from higher to lower locations, we confirmed that the proposed system resulted in a lower elbow position compared with the traditional prosthesis, and the number of blocks transported increased. This significantly reduced the compensatory motion of the elbow and improved the user’s performance compared with the use of a conventional prosthetic hand. This study demonstrates the usefulness of a new myoelectric prosthetic hand that utilizes the residual functions of people with hand deficiencies, which have not been utilized in the past, and the direction of its development.

## Introduction

Myoelectric prosthetic hands are electric prosthetic hands that are operated by acquiring biometric signals, specifically myoelectric potentials, generated during muscle contraction. This prosthetic hand allows people with forearm deficiencies to restore their grasping function by utilizing residual muscle signals. A myoelectric prosthetic hand consists of several key components: a hand that performs the gripping function, a socket that connects the biological and mechanical parts, sensors that detect biometric signals, and a controller that interprets myoelectric potentials to determine the intention of movement.

In daily life, the objects to be grasped have various shapes, necessitating a range of gripping postures. To accommodate this diversity, researchers have developed prosthetic hands with an increased degree of freedom (DOF) in fingers and added wrist joints [1–3]. These highly articulated systems are expected to reduce compensatory movements that occur during gripping [4,5].

However, replicating the 3-DOF of a human wrist joint—flexion and extension, radial and ulnar deviation, and pronation and supination—is challenging for myoelectric prostheses. There are 2 types of challenges: hardware- and control-related challenges. Hardware challenges include the need to introduce the 3-DOF within the limited space of the wrist and develop a wrist mechanism that is durable against external forces. In terms of control, the challenges involve the necessity to interpret and manage the intended movements from sensors, including electromyogram (EMG) signals corresponding to these DOFs. To replicate hardware-related wrist functionality, Legrand et al. [6] managed to achieve pronation and supination movements using a single motor, whereas Damerla et al. [7] enabled palmar and dorsal flexion as well as radial and ulnar deviation using 2 motors. These advancements have enabled more natural wrist movements. In terms of control, Kato et al. [8] used muscular prominence sensors to control palmar and dorsal flexion, reducing compensatory movements through shoulder torque, whereas Bennett and Goldfarb [9] controlled pronation and supination using inertial sensors and demonstrated a reduction in compensatory movements through the task time in the Southampton Hand Assessment Procedure, a type of the hand function test. These studies contribute to a more efficient operation and increased comfort for the users of myoelectric prostheses.

Several people have missing fingers but retain their wrist joints similar to those of non-disabled people [10]. Previous research has addressed this by fixing the wrist to the socket; however, this approach limits the wrist movement and tends to place the prosthetic hand farther away from the body compared with a non-disabled hand. By contrast, if the remaining wrist joint is utilized, the user can intuitively move his/her own wrist, and the prosthetic hand can be used with a feeling of control that is more similar to that of a non-disabled hand.

In this study, we proposed a new myoelectric prosthetic system for individuals without fingers, focusing on 2 aspects: mechanism and control. Mechanically, we proposed dividing the socket into a hand part and a sensor measurement part while preserving wrist joint functionality. In terms of control, we separated the wrist joint signals from the hand signals to ensure that wrist movements did not inadvertently affect hand movements. This allows users to easily and unconsciously achieve their desired joint angles, significantly reducing compensatory movements.

In this field, a myoelectric prosthetic hand that is suitable for patients with residual wrist function has not been developed. This study established a methodology and verified the effectiveness of a myoelectric prosthetic hand that could be used while maintaining the function of the residual wrist. Therefore, this study presents a new direction for the fabrication of myoelectric prostheses.

## Materials and Methods

### Myoelectric prosthesis hand

The custom-made myoelectric prosthetic hand system developed in this study comprises 5 main components: a hand tool, a glove, a control unit, an EMG sensor unit, and a socket interface.

1. Hand tool

We developed a prosthetic hand for individuals who lack fingers. This hand can interpret movement commands from EMG signals that control the opening and closing of artificial fingers. Conventional alternatives, such as Ottobock’s Myobock hand, are too large and heavy (462 g) to suit individuals with finger deficiencies. The developed hand incorporates a bevel gear system (96 g) [11] that allows for the horizontal positioning of the DC motor and space conservation.

2. Glove

Most prosthetic hand gloves are made of vinyl chloride or silicone. These gloves aim not only to enhance the aesthetic appeal, similar to cosmetic prosthetic hand gloves, but also to optimize the grip strength and movement tracking capabilities. We incorporated gloves made from thermoplastic styrene elastomers [12] to address the functional and esthetic considerations.

3. Control unit

The controller measures 35 × 35 × 14 mm and weighs 28 g. We used the SH72546R chipset (Renesas Electronics Corporation, Tokyo, Japan). The chipset operates at a clock speed of 200 MHz, enabling the real-time processing of EMG signals ranging from 0 to 400 Hz. The board was equipped with an analog input for quantizing EMG signals, a digital output for motor control, and a Bluetooth module RN42-I/RM (Microchip Technology Inc., Shanghai, China) for communication with a tablet that instructs hand movements through machine learning. We applied machine learning algorithms to categorize movements using data from the EMG sensors.

4. EMG sensor unit

The sensor features 2 main parts: an electrode and an amplification module. The electrode part is made of a metal woven fabric covered with a conductive silicon layer with 4% and 2.6% carbon powder ECP600JD (Lion Specialty Chemicals Co., Ltd., Tokyo, Japan) and is connected to a gold-plated wire [13]. The module utilizes AD620 circuits (Analog Devices, Inc., Tokyo, Japan) with differential amplification principles for signal amplification. We employed 3 channels to account for the arm circumference and ensure stable control.

5. Socket interface

The socket serves as the mechanical link between the residual limb and the artificial hand. Its dual purpose is to convey movements from the residual limb effectively to the prosthetic hand and relay sensory feedback from the prosthetic back to the residual limb [14].

Individuals with symbrachydactyly often use cosmetic hand prostheses such as disarticulation or partial hand prostheses. In addition, a sleeve-like socket is typically accompanied by the use of a myoelectric prosthetic hand covering the forearm. This situation leads to the loss of the residual function of the user owing to limited hand mobility, necessitating compensatory movements that subsequently decrease their willingness to use the myoelectric prosthetic hand [15]. The system commonly used to control a myoelectric prosthetic hand typically includes either threshold or proportional control, reacts to EMG signals, and recognizes grip actions. However, this approach has limitations in differentiating between various movements. Using the forearm EMG as an input allows only basic open and close commands as outputs. Therefore, it is essential to stabilize the wrist to achieve a stable grip while maintaining the hand position unchanged, which consequently limits the range of motion. When the wrist is immobilized, compensatory movements emerge during the gripping action, similar to the forearm socket.

To facilitate the use of the remaining wrist capabilities, we developed a novel socket and integrated it with an individually adaptive control [16] that allows for multiple-DOF control.

### Proposed socket

**Fig. 1.**
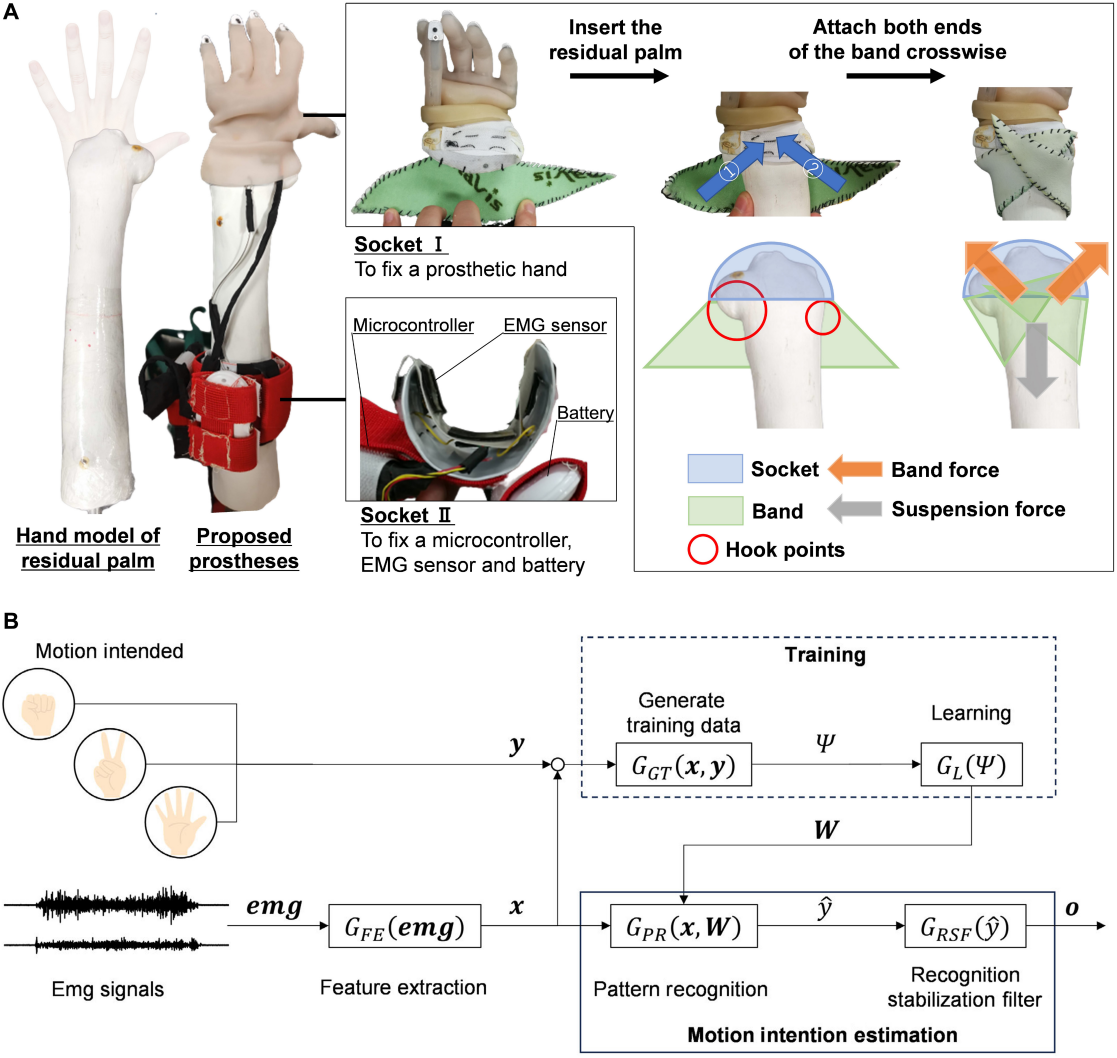
Proposed myoelectric prosthetic system. (A) Overview of the proposed socket type and fixation of the socket; and (B) control system.

The design of the proposed socket is bifurcated into 2 distinct parts to ensure wrist mobility—one for hand stabilization and another for measuring EMG signals—and the developed socket is described as a “separate type.” A schematic of the proposed socket design is illustrated in Fig. 1A. The configuration of socket II includes elements such as an EMG sensor unit, a socket interface, a battery, and a control unit for measuring the EMG activity. The configuration of socket I is simpler and consists solely of the socket interface and the hand component. These sockets were fabricated from the plaster molds of the participants with residual palm. Specifically, they were fabricated by the thermal deformation of acrylic-modified high-impact polyvinyl chloride sheets (Sumitomo Bakelite Co. Ltd., Osaka, Japan). Notably, socket I was designed not to obstruct wrist movement and was, therefore, cut at a point 10 mm from the wrist joint. Additionally, a fixing band (AS ONE Corporation, Osaka, Japan) was sewn onto the socket using a thread. Velcro was used to secure the sockets of the residual limbs. Moreover, to prevent disconnection of the wires between the hand in socket I and the controller in socket II, these sockets are connected with stainless-steel wires, which are shorter than electrical wires. This socket wraps around the palm and is secured by hooking a rubber band over the protrusion located between the wrist and the palm, as shown in Fig. 1A. More importantly, this socket design does not restrict wrist movements or compromise any of the remaining functional capabilities. This design allows the sockets to be customized based on the physical characteristics of the patient, thereby enhancing comfort and functionality. Furthermore, the use of stainless-steel wires plays a crucial role in preventing wire disconnection, thereby improving the durability of the device.

### Application of control methods

When the wrist has freedom of motion while threshold-based controls are applied, unintended grasping actions may occur if the hand moves solely by flexion. Therefore, a system that can discriminate between the intentions of hand and wrist motions is required. In this study, the developed control system employs individuality adaptive control [16], which is a type of pattern recognition control capable of identifying multiple movements based on EMG signals.

The system is divided into 2 processes: a training process for learning EMG patterns and a motion intention estimation process for estimating the intention of motion from the training data in Fig. 1B.

During the training process, the association between exercise intention *y* and measured ***emg*** features was determined. First, when the user teaches the machine regarding motor intention *y*, the EMG data measured by the EMG sensor are transformed by the feature extraction function *G_FE_* into a feature ***x*** suitable for motor intention identification. Next, teacher data *Ψ* = (*x*, *y*) are generated by the teacher data generation function *G_GT_*. These teacher data contain information within a specific period from when feature ***x*** was taught by the user and are buffered by *G_GT_* and then labeled to form a dataset of teacher data *Ψ*. Using *Ψ*, the statistical learning function *G_L_* establishes the relationship between ***emg*** and motion intention *y* and derives the parameters *W* of the pattern recognition function *G_PR_*.

In the motion intention estimation process, the motion intention was estimated from the current EMG data based on the training data. ***emg*** are converted into feature ***x*** by *G_FE_*, similar to the training process. The predicted value of motor intention $\hat{y}$ is then outputted by the pattern recognition function *G_PR_* using the preadjusted parameter *W*. $\;\hat{y}$ is selected from the set of motion intention *y* inputted into the training process. The recognition stabilization filter function *G_RSF_* outputs the motor intention *o* from the input $\hat{y}$. If the motor intentions estimated from the EMG signals contain misidentification results, hand movements will not be stable. Therefore, *G_RSF_* adjusts the output based on the proportion of motor intention estimates within a specific period. *o* is selected from a set of motor intentions *y* and $\hat{y}$.

This control system ensures that flexing and extending the palm are distinguished from actions related to opening or closing the hand, thus allowing the wrist to operate independently and leverage the residual wrist function for control.

### Signal processing

Biological signals are measured using EMG sensors [13]. In this study, the EMG signals were filtered using a bandpass filter from 1 to 1,000 Hz and a 50-Hz notch filter present within the EMG sensor amplifier and then amplified 80,000 times through a differential amplification circuit. These signals were measured at a sampling frequency of 2,000 Hz. A 50-Hz digital high-pass filter and a Hann window with a frame size of 256 were employed to divide the measured signals into 8 regions ranging from 0 to 400 Hz for each sensor using a fast Fourier transform. The control method used in this study utilized pattern recognition control using artificial neural networks. Because 3 channels of the EMG sensors were used, the feature value amounted to 24 data points.

## Experiment

In this section, we describe the experimental procedures for evaluating compensatory movements when using myoelectric prosthetic hands. The magnitude of compensatory movements was calculated based on the trunk angle.

### Participant information

The participant pool consisted of 3 non-disabled men in their 20s (participants A, B, and C) and one 11-year-old male participant with symbrachydactyly (participant D). The 3 able-bodied participants were right-handed, and EMG data were collected from their right forearms, whereas for the participant with symbrachydactyly, data were gathered from the left forearm. We measured the EMG signals using an EMG sensor specifically designed for this study, featuring 3 channels. CH1 was positioned on the extensor carpi radialis brevis muscle, CH2 was positioned on the ulnar carpal flexor muscle, and CH3 was positioned on the flexor carpi radialis muscle, with the body GND placed on the ulna. This study was approved by the University of Electro-Communications Ethics Committee for Human Experiments (No. 10006(7)). All participants provided informed consent.

### Task

When the wrist is immobilized through a socket, shoulder movements assume the roles normally performed by the wrist, often resulting in increased lifting of the elbow [17]. Preliminary tests verified that these compensatory movements engage the entire body.

Movements that involve substantial wrist activity include reaching upward to grasp objects at heights. The task in this study was to transfer a set of 10 cubes, each measuring 2.5 × 2.5 × 2.5 cm^3^, from a high starting point to a low ending point. The field used in this study was constructed with the start and end areas of 125 × 125 mm. The start area was positioned higher than the end area, with a height difference of 300 mm. The gap between the 2 areas was 500 mm. These areas were placed 200 mm away from the participants, providing an optimal configuration for observing their movements and responses. Furthermore, a camera was installed in front of the participants at a distance of 1,700 mm. We not only monitored the elbow positions but also observed both shoulder locations to calculate the trunk angle. The magnitude of compensation was assessed by integrating the absolute trunk tilt values. We gauged the effectiveness of the prosthetic hand by measuring the extent to which the task could be completed within a 30-s video segment.

### Upper limb position measurement

The position of the upper limb was measured using the motion capture system MediaPipe (Google, California, USA) [18]. The motion capture system consists of a single webcam connected to a laptop and can be used to measure 33 anatomical landmarks. In this study, 4 anatomical landmarks (both shoulders and elbows) were measured. The trunk angle was calculated using the position data obtained using the motion capture system. The experiment was conducted on a Windows 10 operating system equipped with an Intel Core i7-8750H (2.20 GHz) processor, 16-GB DDR4 RAM, 256-GB SSD, and an NVIDIA GeForce GTX 1060. The positions of both shoulders and the right elbow, with reference to the top-left corner of the screen, were measured at 0.11-s intervals. Variability in the count of these measurements reached up to a 1% error owing to the progress of image processing.

### Evaluation of compensatory movement

The distortion level *f*_(*θ*)_ was defined by Eq. 1 as the total number of trunk angles in a single session because compensatory movements cannot be evaluated based only on the body’s inclination at that moment but are evaluated in the form of energy. In this study, *n* represents the total number of frames in a task, as measured using MediaPipe; and *θ_k_* indicates the trunk angle (degrees) at the *k*th frame. Therefore, the magnitude of compensatory motion can be evaluated.

DΘ=∑k=0nθk (1)

$$\Theta =\left\{\theta_{0},\theta_{1},\cdots ,\theta_{k},\cdots ,\theta_{n}\right\}$$

### Instruction procedure

When the wrist joint was in a pronated position, we collected EMG data in the rest, grip, and open states. These signals are associated with the movement of each hand. However, as flexion and extension movements influence hand actions, we trained the system to exclude signals related to these movements from hand actions.

### Adapting the myoelectric prosthetic hand

**Fig. 2.**
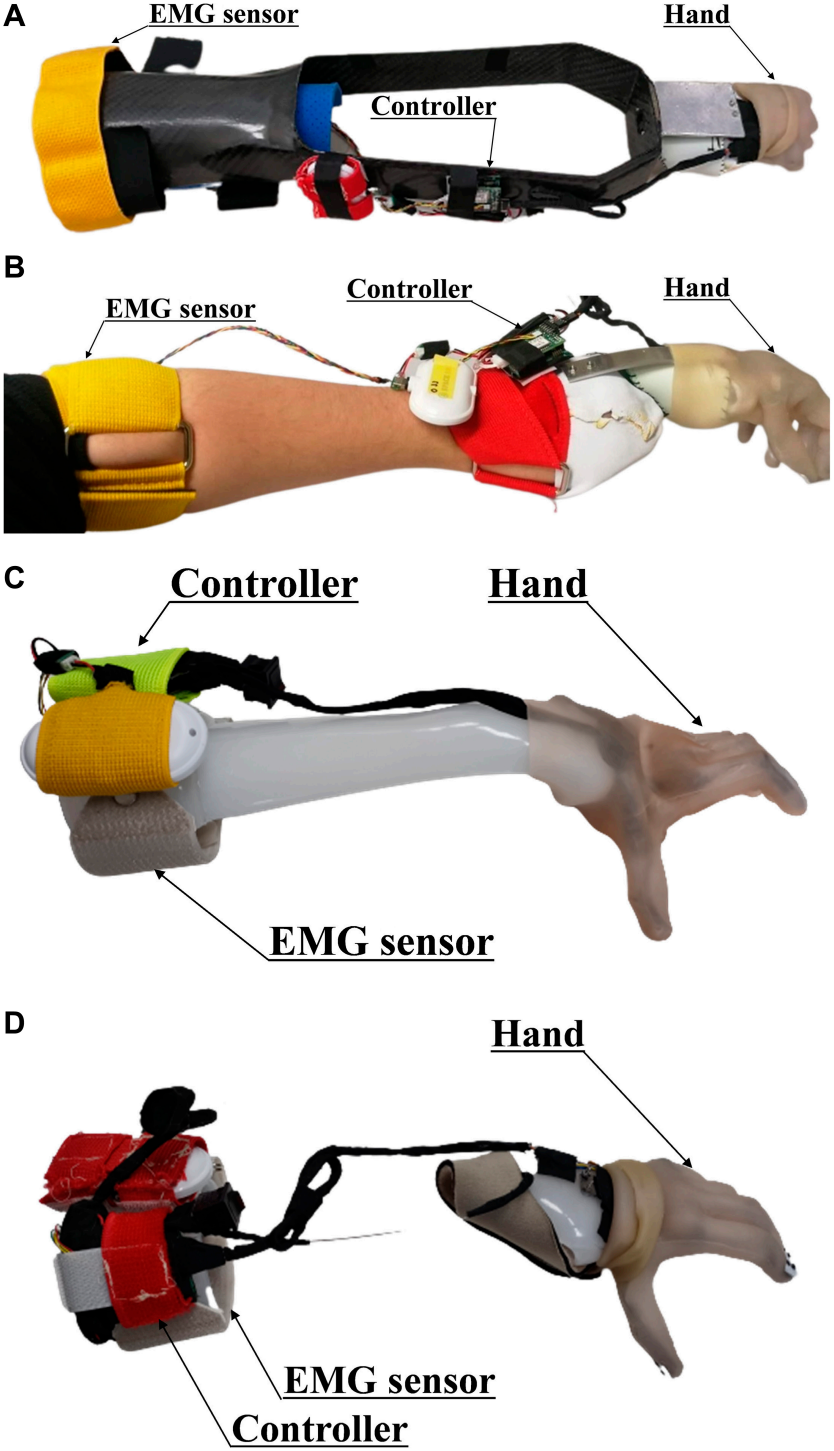
Conventional and developed myoelectric prosthetic hands (the electric prosthetic hand is attached to the able-bodied socket. The separate-type prosthesis is composed of 2 components: a white socket and a yellow EMG sensor band to free the wrist area without disturbing flexion and extension). (A) Conventional-type and (B) separate-type prostheses for non-disabled participants. (C) Conventional-type and (D) separate-type prostheses for the participant with symbrachydactyly.

The components of the EMG prosthesis utilize a system based on the structure of the EMG prosthesis, as described in the “Materials and Methods: Myoelectric Prostheses” section. Because the conventional socket does not allow the use of wrist functions, we adopted a forearm socket for non-disabled individuals, as shown in Fig. 2A, to ensure that the wrist movements do not affect the prosthetic hand. As this socket is fixed to the forearm, the prosthetic hand is not affected by wrist movements, and its position is manipulated solely by forearm movements. By contrast, the developed socket shown in Fig. 2B utilizes the mechanism described in the “Materials and Methods: Myoelectric Prostheses” section. The wrist angles of the hand tool in each socket were set to minimize compensatory movements.

In the experiments, as depicted in Fig. 2, the hand tools for both non-disabled participants and the participant with symbrachydactyly were interchangeable to standardize the hand performance. Given that the block used in the tests weighed 13 g, hand stiffness did not influence the tracking of the tip position.

In the experiment, we used the newly proposed socket and control system. We then quantitatively compared the ease of executing a grasping action between the proposed and conventional sockets.

## Results and Discussion

### Magnitude of compensatory movements and elbow trajectory of non-disabled participants

**Fig. 3.**
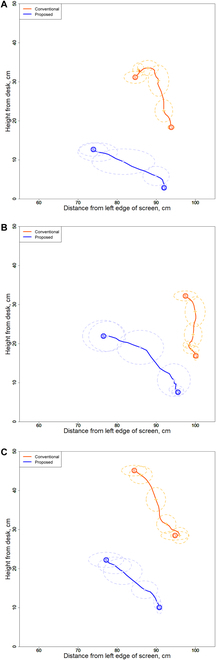
Elbow paths with conventional and separate-type prostheses for non-disabled participants (A) A, (B) B, and (C) C.

We evaluated the magnitude of compensatory shoulder motion owing to the elbow position in non-disabled participants. Figure 3 shows the average trajectory of the elbow for 10 trials in each socket. The data in each trial were aligned to derive the average trajectory of the elbow. Therefore, we used the trial with the largest amount of data as the standard and linearly interpolated the lack of data to unify them. Here, *S* represents the start position and *G* represents the goal position. All data were divided into 5 equal parts, and the points are indicated by crosses; the standard deviation at that point is indicated by an ellipse.

Table 1 lists the average elbow heights from the tabletop for all participants.

**Table 1.** Average height from the tabletop using conventional and separate-type prostheses for non-disabled participants

Participant		A			B			C		
		Conventional	Proposed	Difference	Conventional	Proposed	Difference	Conventional	Proposed	Difference
Base point	Height (cm)	34.4	13.7	20.6	32.3	22.8	9.50	45.1	22.2	22.9
	SD	1.87	1.71		1.37	3.73		1.38	1.94	
End point	Height (cm)	18.3	2.6	1.57	16.6	7.24	9.37	27.6	10.0	17.6
	SD	0.57	0.51		0.906	1.099		1.37	1.58	

For participants A and B, the trajectory of the elbow was observed to move once to the left when it was lowered from above. The reason for this observation was that the elbow position was shifted to the hand side when the skeleton was detected by machine learning inference because the hand tool was judged to be a hand in MediaPipe image recognition.

Movement was observed along a longitudinal trajectory in all participants using the conventional-type prosthesis. When using the separate-type prosthesis, the orbit was generated at a lower position than that generated when using the conventional-type prosthesis and was longer in the transverse direction.

The primary reason for the large longitudinal displacement of the elbow trajectory in the conventional socket type is the mounting angle of the hand tool. This angle is set to reduce the compensatory motion when picking up tabletop objects. When grasping an object placed at a higher position using the hand tool, as shown in Fig. 3A, the shoulder is flexed and abducted to adjust the angle between the hand tool and the object to be grasped. This increases the elbow position. By contrast, when grasping an object placed at a lower position, the shoulder is extended and adducted to adjust the position of the hand tool. The elbow trajectory is predominantly a longitudinal displacement. In the separate socket type, the elbow trajectory is linear because wrist flexion and extension are used to adjust the angle of the hand tool. Therefore, the magnitude of longitudinal displacement of the elbow trajectory can be used to determine the degree of compensatory movement in a field task with different elevations.

The reduction in the elbow position in the separate socket type revealed a reduction in shoulder abduction motion. From this point, a reduction in compensatory motion was indicated because the compensatory motion of the wrist significantly affects the amount of shoulder abduction and, thus, the elbow position.

The elbow position when using the separate socket type (Table 1) was 9 to 20 cm lower than that when using the conventional socket type. Thus, compensatory movements of the shoulder owing to the elbow position were reduced in all participants.

Therefore, the elbow position was lowered when the separate socket type was used. As a result, the utilization of the remaining wrist joint was indicated by a reduction in shoulder abduction motion.

### Evaluation of compensatory movement through the body for non-disabled participants

**Fig. 4.**
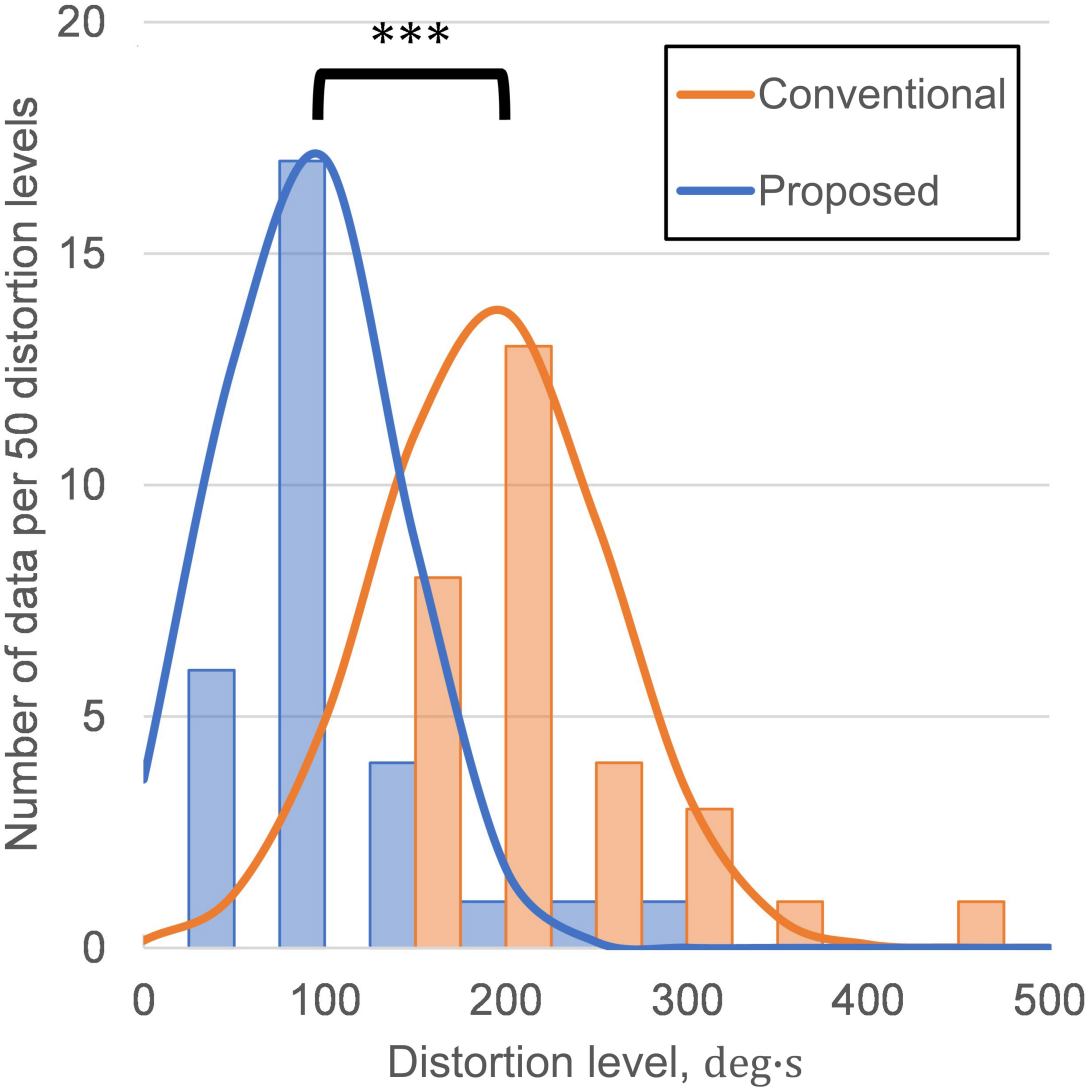
Relationship between the distortion level and the number of data per 50 distortion levels for participant D with symbrachydactyly.

The histograms are aggregated by the magnitude of the distortion level for each trial for the conventional and separate socket types in Fig. 4. The rank width is 50 distortion levels. The horizontal axis shows the magnitude of the distortion level, and the vertical axis shows the number of data per 50 distortion levels. The curves show the normal distribution of the distortion level for each socket.

**Fig. 5.**
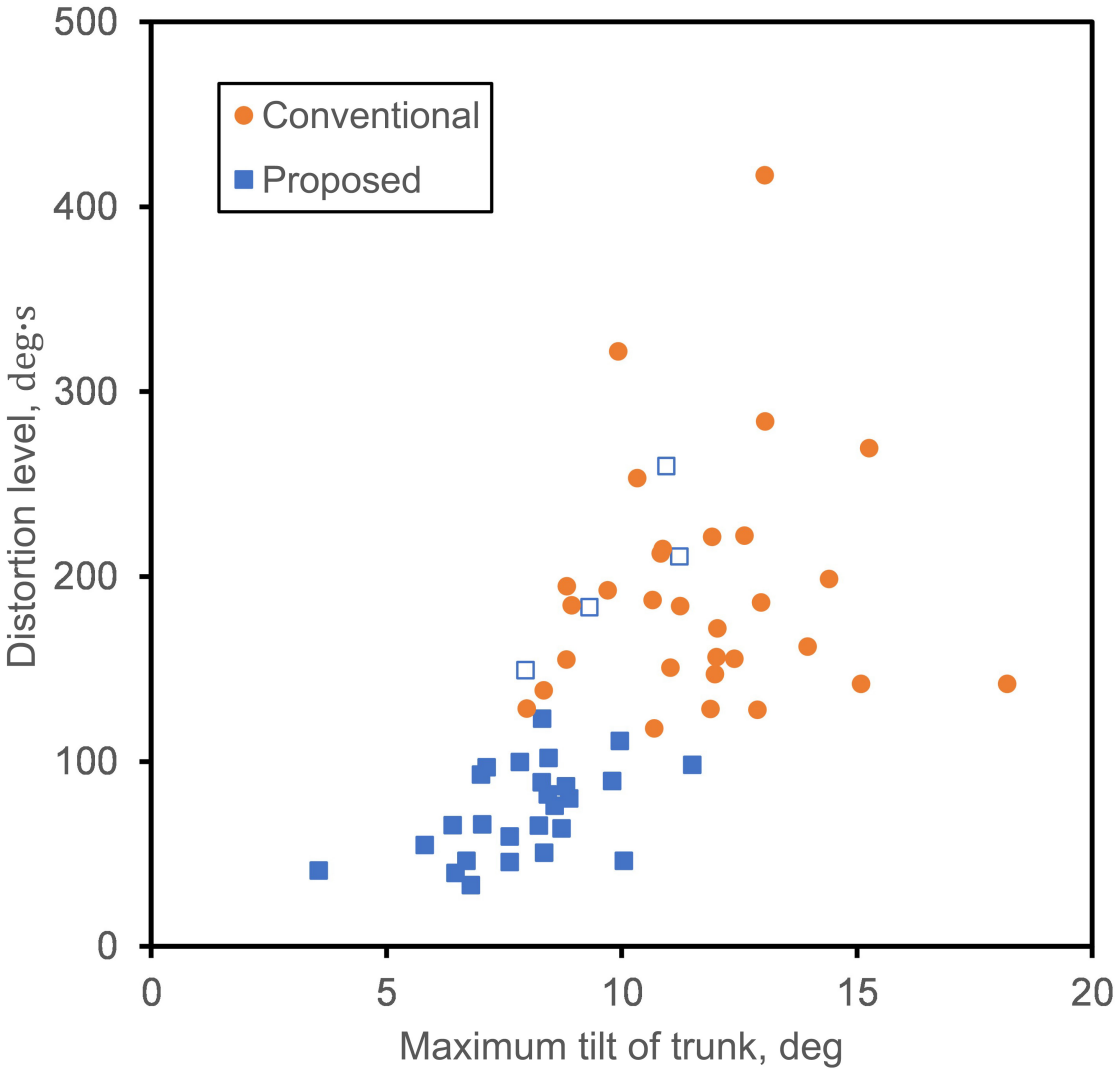
Relationship between the distortion level and the number of data per 50 distortion levels for non-disabled participants.

The differences in distortion levels were analyzed for each socket type (Fig. 5). In the distribution of the distortion level, when using the conventional and separate socket types, the peaks appeared at 210 deg · s and 90 deg · s, respectively. It is evident that the distortion level is lower for the separate socket type at the peak values.

The value was 192 deg · s (standard deviation: 64.0) for the conventional type and 90.2 deg · s (standard deviation: 51.0) for the separate type. We conducted a *t* test and observed a significant difference between the conditions [*t*(29) = 7.75; *P* < 0.05]. Because a significant difference was observed between the socket types, we considered the compensatory motion to be greater with the use of the conventional socket type with a higher distortion level. It is evident that the grasping movements in cases where the wrist joint is not disabled involve fewer compensatory movements. Therefore, we inferred from the above data that a separate socket type with reduced compensatory movements effectively utilizes the remaining wrist joints.

**Fig. 6.**
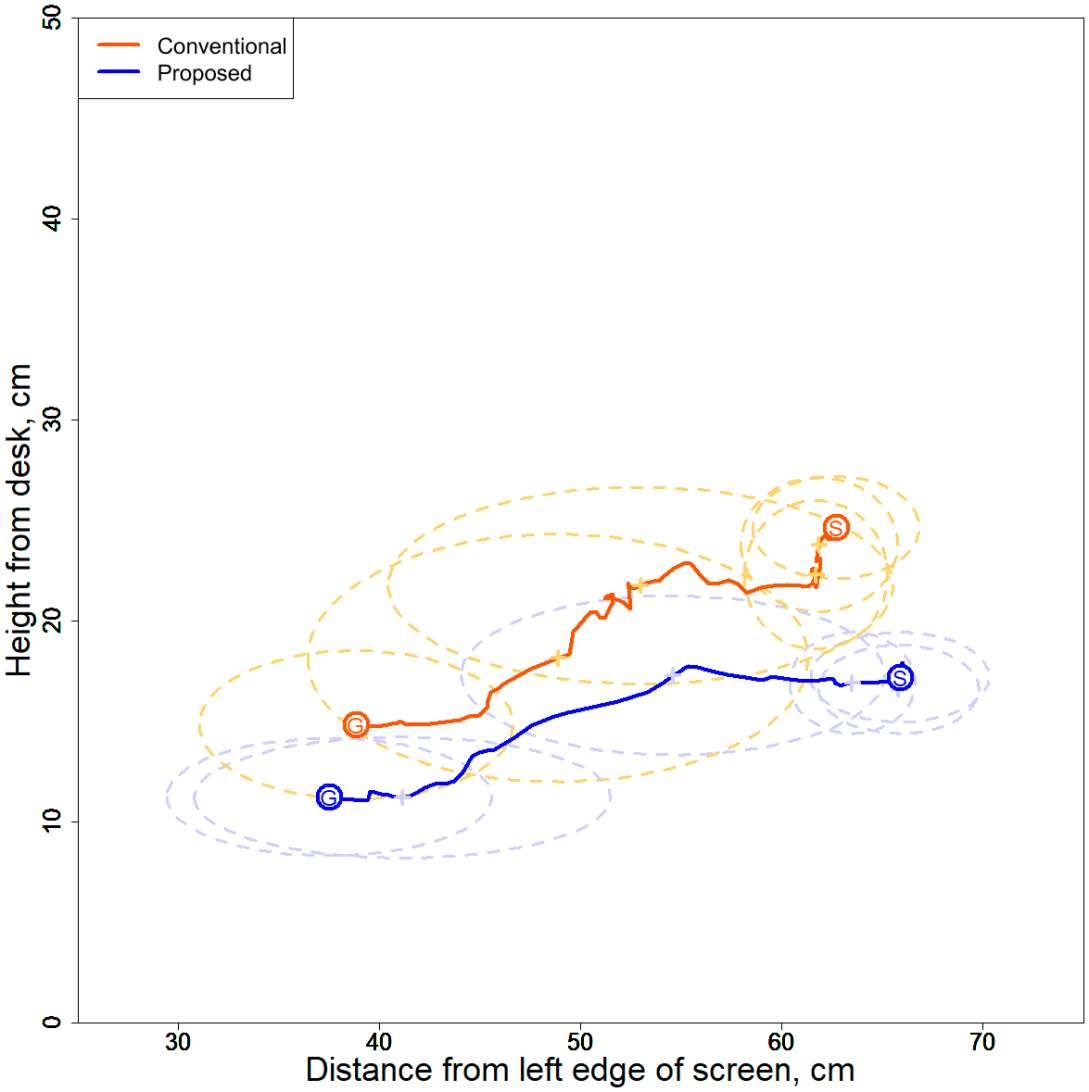
Relationship between the maximum tilt of the trunk and the distortion level for non-disabled participants.

The relationship between the maximum trunk angle and the distortion level in each experiment is shown in Fig. 6. The smaller the maximum angle, the lower the distortion level. The distribution was divided between the top and the bottom, and the distortion obtained with the separate socket type was low, indicating that the compensatory movement was minor. Movements that required longer than 4 s with the separate socket type are represented by hollow squares. The distortion level increased over time, even when the maximum trunk angle was small. Therefore, the distortion level indicates the burden on the users.

### Measurement performance of each socket type for non-disabled participants

The number of blocks grasped and carried, as measured in 30 s, was 4.6 with the conventional socket type and 5.6 with the separate socket type. These results indicate that the separate type performed better than the conventional type because the latter required more time as the body acquired an unreasonable position during grasping. The performance was affected by the fact that the separate socket type moved more linearly from the orbit than the conventional socket type.

### Magnitude of compensatory movements and elbow trajectory of the participant with Symbrachydactyly

**Fig. 7.**
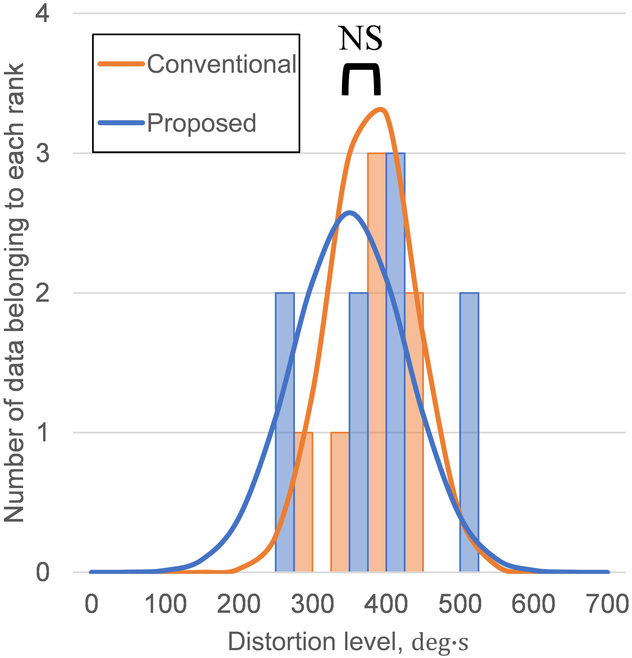
Elbow paths with the use of conventional and separate socket types for participant D with symbrachydactyly.

We evaluated the magnitude of compensatory shoulder motion owing to the elbow position in participant D with symbrachydactyly. Figure 7 shows the average trajectory of the elbow during 10 runs with each socket type. The same procedure was used to process the data, as shown in Fig. 3. Table 2 lists the average elbow height of participant D from the tabletop.

**Table 2.** Average elbow height from the tabletop when using conventional and separate socket types for participant D with symbrachydactyly

		Conventional	Proposed	Difference
Base point	Height (cm)	26.4	20.1	6.32
	SD	2.58	2.78	
End point	Height (cm)	14.8	11.25	3.60
	SD	3.68	2.92	

As participant D operated the prosthetic hand with his left hand, as shown in Fig. 7, the elbow trajectory with all socket types was diagonally upward to the right, differing from the direction observed in non-disabled participants (Fig. 3). With the use of the separate socket type, the elbow trajectory was in a low position, similar to that observed in non-disabled participants. However, the trajectory was more erratic than that noted in non-disabled participants. This was because of the problem of image recognition using MediaPipe. Similar to that observed in non-disabled participants, when the hand tool was moved, the positions of the hand tool and the actual hand were alternately switched, resulting in a noisy trajectory. The elbow positions at the base and endpoints were inferred correctly because the hand tool position had not moved.

The elbow position when using the separate socket type (Table 2) was 3 to 6 cm lower than that when using the conventional socket type. Thus, the compensatory movements of the shoulder owing to the elbow position were reduced in participant D.

The aforementioned factors confirmed that the elbow position is reduced when using the separate socket type, and the developed system allowed participant D to utilize the remaining part of the wrist function.

### Evaluation of compensatory movement throughout the body for the participant with symbrachydactyly

Table 3 lists the distortion level on each socket type for participant D. We eliminated data in which the user required more than 10 s to operate the prosthetic hand. The histograms are aggregated by the magnitude of the distortion level for each trial for the conventional and separate socket types in Fig. 4. The rank width is 50 distortion levels. The horizontal axis shows the magnitude of the distortion level, and the vertical axis shows the number of data per 50 distortion levels. The curves show the normal distribution of the distortion level for each socket.

**Table 3.** Time and compensatory movements per one execution in participant D with symbrachydactyly

Conventional			Proposed		
Time (s)	Degree (deg)	Distortion (deg s)	Time (s)	Degree (deg)	Distortion (deg s)
4.20	15.2	38.1	4.68	15.1	351

The differences in distortion levels were analyzed for participant D with the use of each socket type (Fig. 4). The value was 381 deg · s (standard deviation: 57.6) for the conventional type and 351 deg · s (standard deviation: 77.5) for the separate type. The *t* test results showed no significant differences between the socket types [*t*(14) = 0.845; *P* = 0.206]. As a result, the mean difference in the distortion level of participant D between the conventional and separate socket types was smaller than that of non-disabled participants. It is possible that participant D used the wrist joint well. Participant D usually used the conventional socket type, not the separate socket type, and learned to grasp compensatory movements while using a hand. In addition, the additional 2 DOFs for wrist flexion/extension of the separate socket type increased the difficulty of the operation compared with the use of the conventional socket type, which may have increased the time required for each operation. Therefore, there were no significant differences between the 2 socket types.

The separate socket type required a longer time per exercise and had a lower distortion level than the conventional socket type. The distortion level is the total value of the trunk tilt per exercise, as shown in Eq. 1. However, this result indicated that the overall torso angle was smaller during exercise. Therefore, we confirmed that the separate socket type had a lower burden of use than the conventional socket type.

Even though prosthetic hands with separate socket types are not normally used, compensatory movements tend to be reduced when compared by time and distortion level, suggesting that the wrist is used even without training. Therefore, the compensatory movement of the separate socket type is further reduced by developing a training method that separates the opening/closing of the hand and wrist flexion/extension.

### Measurement performance of each socket type for the participant with symbrachydactyly

The number of blocks grasped and carried, as measured in 30 s, was 3 with the use of the conventional socket type and 3 with the use of the separate socket type. Unlike the performance results of non-disabled participants, the number of blocks that participant D was able to carry was the same with the use of both socket types. This could be attributed to the increased operational difficulty, as explained in the “Evaluation of compensatory movement throughout the body for the participant with symbrachydactyly” section.

## Conclusion

When operating a prosthetic hand, it is advantageous for users to exert minimal energy. The use of conventional sockets often results in compensatory movements, and this phenomenon is observed even in participants with symbrachydactyly who retain partial wrist functionality. Such movements increase the stress on the user.

The aim of this study was to develop a socket optimized for participants with symbrachydactyly, capitalizing on their residual wrist capabilities. We conducted a quantitative evaluation comparing the ease of the grasping motion using our newly designed separate type socket with that of the conventional socket. The innovative design of the developed socket facilitates unobstructed wrist movement by separating the sections for hand attachment from the EMG sensor, thereby minimizing the need for compensatory elbow movements. Our findings indicated a notable decrease in compensatory movements among non-disabled participants using this design. Furthermore, a reduction in compensatory movements was observed in the participant with symbrachydactyly, particularly in terms of elbow positioning and time–distortion level relationships. In addition, the manipulation performance of non-disabled participants with the separate socket improved, whereas no change was observed in the performance of the participant with symbrachydactyly who did not normally use the separate socket. However, a decrease in compensatory movements suggests that the performance of participant with symbrachydactyly may improve with prolonged use.

In view of the above findings, this study demonstrates the usefulness of a new myoelectric prosthetic hand that utilizes the residual functions of people with hand deficiencies, which have not been utilized in the past, and the direction of its development.

This study involved only 3 non-disabled participants and one participant with symbrachydactyly. Therefore, this limited dataset may not enable a comprehensive analysis. Furthermore, the unfamiliarity of the participants with the newly designed sockets may have prevented them from fully realizing their benefits. Consequently, future studies should include a larger cohort of participants with symbrachydactyly and develop training methods that better leverage the advantages of these innovative sockets.

## Data Availability

The data used to support the findings of this study are available from the corresponding author upon reasonable request.
